# Genetic Analysis of Pitt–Hopkins Syndrome Caused by a Novel Splicing Variant (c.1146+3A>T) in the *TCF4* Gene

**DOI:** 10.1002/mgg3.70239

**Published:** 2026-05-28

**Authors:** Wenlong Shen, Yan Zhang, Junjie Wu, Jue Zhao, Yaer Lv, Xiaohua Tang

**Affiliations:** ^1^ Hangzhou Medical College Hangzhou Zhejiang China; ^2^ Laboratory Medicine Center, Department of Genetic and Genomic Medicine Zhejiang Provincial People's Hospital, Affiliated People's Hospital, Hangzhou Medical College Hangzhou Zhejiang China

**Keywords:** increased nuchal translucency, Pitt–Hopkins syndrome, prenatal diagnosis, splicing variant, *TCF4*

## Abstract

**Objective:**

Pitt–Hopkins Syndrome (PTHS) is a rare genetic disorder primarily caused by *TCF4* mutations and involves developmental, intellectual, and physical changes in children. Increased nuchal translucency (NT) has not been associated with *TCF4* mutations or PTHS. Here we study the connection between increased NT and a c.1146+3A>T mutation in the *TCF4* gene.

**Methods:**

The genetic basis of increased NT in an early pregnancy fetus was investigated by family trio clinical exome sequencing (CES) using fetal amniotic fluid and parental peripheral blood samples. The candidate variant was validated by Sanger sequencing. The impact of the variant on transcription was assessed using a minigene assay.

**Results:**

Early ultrasound revealed an NT measurement of 3.5 mm in fetus B of a twin pregnancy. CES identified a de novo heterozygous c.1146+3A>T variant in intron 14 of *TCF4*, confirmed by Sanger sequencing. In vitro minigene experiments showed that the mutation disrupted *TCF4* mRNA splicing, resulting in exon 14 skipping and a truncated transcript.

**Conclusion:**

Our results identified c.1146+3A>T as a novel splicing variant of the *TCF4*. The *TCF4* c.1146+3A>T mutation may underlie increased NT in early pregnancy, suggesting that increased NT could be an early intrauterine sign of PTHS.

## Graphical abstract

A novel pathogenic splicing variant (TCF4c.1146+3A>T) was identified in a fetus with increased nuchal translucency. The variant disrupts normal splicing, causing exon 14 skipping and protein truncation, which is consistent with Pitt–Hopkins Syndrome.

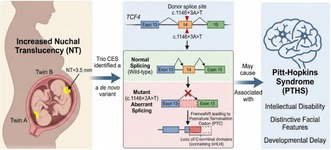

## 1 Introduction

Nuchal translucency (NT) refers to a transient accumulation of fluid at the posterior aspect of a fetal neck. NT appears on ultrasonography as an anechoic space between the skin and the underlying soft tissues. During weeks 11 through 14 of gestation, the cervical lymphatic vessels gradually establish communication with the jugular venous system (Jiang and Li 2024). However, before this connection is fully formed, a small amount of lymphatic fluid may accumulate in the cervical region, giving rise to the NT. This is considered a normal physiological phenomenon during early fetal development, and in most normal fetuses, the NT typically regresses after week 14 of pregnancy. The NT thickness is a routine ultrasonographic marker in first‐trimester prenatal screening, and an NT greater than 2.5 mm is generally considered a risk factor for adverse fetal outcomes (Yin et al. 2022), encompassing a broad spectrum of diseases ranging from chromosomal abnormalities and structural anomalies to a variety of genetic syndromes, including Noonan syndrome, skeletal dysplasias, and neurodevelopmental disorders (Atallah et al. 2025).

Pitt–Hopkins syndrome (PTHS) is a severe neurodevelopmental disorder characterized by profound intellectual disability, craniofacial abnormalities, distinctive facial features, and generalized hypotonia in children. Some individuals with PTHS may also present with epilepsy, myopia, and episodic hyperventilation. Nevertheless, to our knowledge, increased NT has not been reportedly associated with PTHS, since it is a disorder usually found in children. The primary disease‐causing gene for PTHS is *TCF4* (transcription factor 4), and the disorder is inherited in an autosomal dominant manner.

With the rapid advancement of high‐throughput sequencing, clinical exome sequencing (CES) has become an important tool in prenatal genetic evaluation. This approach improves the identification rate of underlying genetic causes and helps genetic counseling (Yavas et al. 2025). CES has revealed variants associated with conditions such as Noonan syndrome and various skeletal dysplasias, expanding the prenatal phenotypic spectrum of known genes and uncovering novel gene–phenotype associations (Atallah et al. 2025). Importantly, CES can detect early genetic links to disorders, including neurodevelopmental conditions that typically present postnatally, providing valuable insights for prenatal diagnosis and clinical management (Baris and Yavas 2025).

In this study, we presented one of the fetuses (fetus B) from a twin pregnancy, who exhibited an increased NT. In the amniotic fluid samples of this fetus, we identified a de novo pathogenic splicing variant, c.1146+3A>T, in the PTHS‐causing *TCF4* gene, thus revealing a novel connection between increased NT and *TCF4* gene mutation or a potential PTHS.

## 2 Materials and Methods

### 2.1 Samples

A twin pregnancy was enrolled in the Zhejiang Provincial People's Hospital in March 2023. The pregnant woman had two frozen embryos transplanted in the hospital, and both survived. It's a dichorionic diamniotic twin pregnancy. The first‐trimester prenatal ultrasonography showed an NT measurement of 1.9 mm in fetus A and 3.5 mm in fetus B, respectively. For prenatal diagnostic purposes, amniotic fluid from fetus B and peripheral blood samples from both parents were collected for trio‐based clinical exome sequencing (CES). This study was approved by the Medical Ethics Committee of Zhejiang Provincial People's Hospital (Approval No. QT2025105).

### 2.2 Methods

#### 2.2.1 Genomic DNA Extraction

Two milliliters of amniotic fluid from the fetus and 2 mL of peripheral blood from each parent were collected in EDTA anticoagulant tubes. Genomic DNA was extracted from amniotic fluid and peripheral blood using a commercial tissue/blood genomic DNA extraction kit (Kaishuo Biotech, Xiamen, China). DNA concentration and purity were assessed using a NanoDrop ultraviolet spectrophotometer (Thermo Fisher Scientific).

#### 2.2.2 Clinical‐Exome Sequencing

Genomic DNA from the fetus and its parents was fragmented, end‐repaired, adaptor‐ligated, amplified, and purified. DNA libraries were prepared using the Freyseq Clinical Exome Capture Kit and sequenced on an Illumina NextSeq 550DX high‐throughput sequencing platform. The assay targeted exonic regions and flanking intronic sequences (±20 bp) of 7099 clinically relevant human genes. After quality control, sequencing reads were aligned to the human reference genome hg19 (GRCh37) using the Burrows‐Wheeler Aligner. Variant annotation and filtering were performed using public databases, including dbSNP, ExAC, HGMD, ClinVar, OMIM, and gnomAD to identify candidate single‐nucleotide variants as well as insertion and deletion variants.

#### 2.2.3 Sanger Sequencing Validation

PCR primers flanking the *TCF4* c.1146+3A>T variant were designed using Primer3Plus for Sanger sequencing validation. The forward primer sequence was 5′‐TTGCATGAAGGTCTAACAAAATCCC‐3′, and the reverse primer sequence was 5′‐TGGAGAGTAAAGGAGACTGAACAAG‐3′. Genomic DNA from the fetus and its parents was amplified by PCR, followed by purification of PCR products using a shrimp alkaline phosphatase (SAP) enzyme mixture. Sequencing reaction was performed using BigDye terminator chemistry, and the products were purified using a sodium acetate–ethanol method before capillary electrophoresis. Sequencing data were analyzed using SnapGene software and compared with reference sequences from GenBank to confirm the variation.

#### 2.2.4 Minigene Splicing Assay

The recombinant plasmids pcMINI‐wt and pcMINI‐mut were constructed based on a normal control human DNA and the fetus B amniotic fluid genomic DNA, respectively. The genomic DNA was used as a template to amplify the target region using two rounds of nested PCR. The primer pairs were F1 (5′‐GAGTCCCAAAAAGGGAGTTG‐3′) and R1 (5′‐AAACCTCTTGGAGTGCATGC‐3′) for the first round, and F2 (5′‐GCTATTTTCACACCGACGGC‐3′) and R2 (5′‐GCAGATTAATTGGGCTGGGT‐3′) for the second round. The second‐round PCR product was used as a template to generate wild‐type and mutant *TCF4* fragments (1404 bp), respectively. The pcMINI vector containing the ExonA–IntronA–MCS–IntronB–ExonB cassette and the target DNA fragments were subjected to restriction digestion, ligation, transformation, and clone validation. Briefly, both the vector and DNA fragments were digested with restriction enzymes KpnI (Yeasen, China) and NotI (Yeasen, China) at 37°C for 2 h, followed by agarose gel electrophoresis and purification of the target fragments. The purified fragments were then ligated into the linearized vector and incubated at 4°C overnight. The ligation products were transformed into DH5α competent cells and cultured overnight at 37°C. Positive clones were validated by Sanger sequencing.

HEK‐293T and HeLa cells in the exponential growth phase were harvested and dissociated using 0.25% trypsin, then seeded into six‐well plates. Transfection was performed when cell confluency reached approximately 60%–70% the following day. Transfections were carried out using Lipofectamine 3000 reagent (Invitrogen) according to the manufacturer's instructions. Briefly, 2.5 μg of either pcMINI‐wt or pcMINI‐mut plasmid DNA and 5 μL of P3000 reagent were diluted in Opti‐MEM I reduced‐serum medium to a final volume of 125 μL, gently mixed, and incubated at room temperature for 5 min. In parallel, 7.5 μL of Lipofectamine 3000 reagent was diluted in Opti‐MEM I to a total volume of 125 μL and incubated for 5 min at room temperature. The two solutions were then combined, gently mixed, and incubated to allow formation of lipid–DNA complexes. The resulting complexes were added dropwise to HEK‐293T and HeLa cells. Cells were incubated for 48 h post‐transfection before subsequent analyses.

The total RNA was extracted 48 h after transfection and reverse‐transcribed into cDNA. The RT‐PCR amplification was performed using primers pcMINI‐F (5′‐CTAGAGAACCCACTGCTTAC‐3′) and pcMINI‐R (5′‐GCCCTCTAGACTGGTCATTCCGGCTC‐3′), followed by Sanger sequencing. The splicing pattern of the mutant was compared with the wild‐type to assess the impact of the c.1146+3A>T variant on *TCF4* mRNA splicing.

#### 2.2.5 Variant Pathogenicity Classification

The pathogenicity of the identified variant was evaluated according to the standards and guidelines for the interpretation of sequence variants: a joint consensus recommendation of the American College of Medical Genetics and Genomics (ACMG) and the Association for Molecular Pathology (Richards et al. 2015).

## 3 Results

### 3.1 The Genetic Findings in Fetus B

**FIGURE 1:**
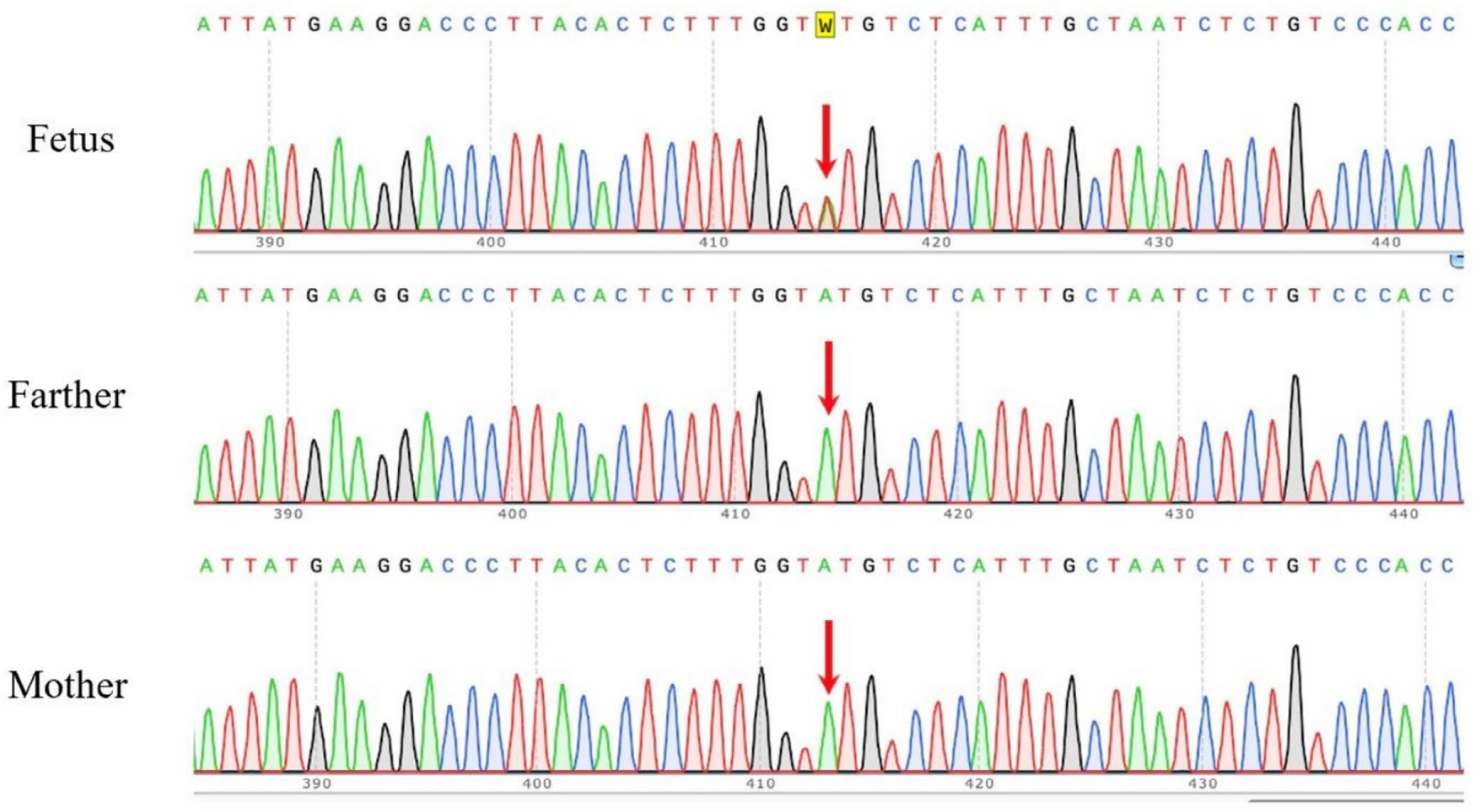
Sanger sequencing validated that the Fetus B carried a heterozygous de novo mutation of *TCF4* c.1146+3A>T. The c.1146+3 locus was highlighted by a downward red arrow.

Trio‐based clinical‐exome sequencing was performed using genomic DNA extracted from the amniotic fluid of fetus B and peripheral blood samples from both parents. During the ultrasound examination, we found that the NT value of fetus A was 1.9 mm. The NT value of fetus A was within the normal range and there was no abnormal prenatal phenotype. Thus, the clinical recommendation was not to take amniocentesis and genetic testing with fetus A. A heterozygous variant, c.1146+3A>T, was identified in intron 14 of the *TCF4* gene (NM_001083962.1) in fetus B, whereas not in the parental samples. Sanger sequencing further confirmed that this de novo variant was present exclusively in the fetus and absent in both parents (Figure 1).

### 3.2 Detection of Copy Number Variants

Based on CES, no pathogenic or likely pathogenic copy number variations (CNVs) involving two or more consecutive exons related to the clinical background of the fetus B were detected in the amniotic fluid sample. In addition, we also conducted a CNV‐seq assay in fetus B. We found that no meaningful CNV variants, i.e., duplication variants greater than 1 Mb or deletion variants greater than 0.5 Mb, were associated with fetus B.

### 3.3 Minigene Splicing Analysis

**FIGURE 2:**
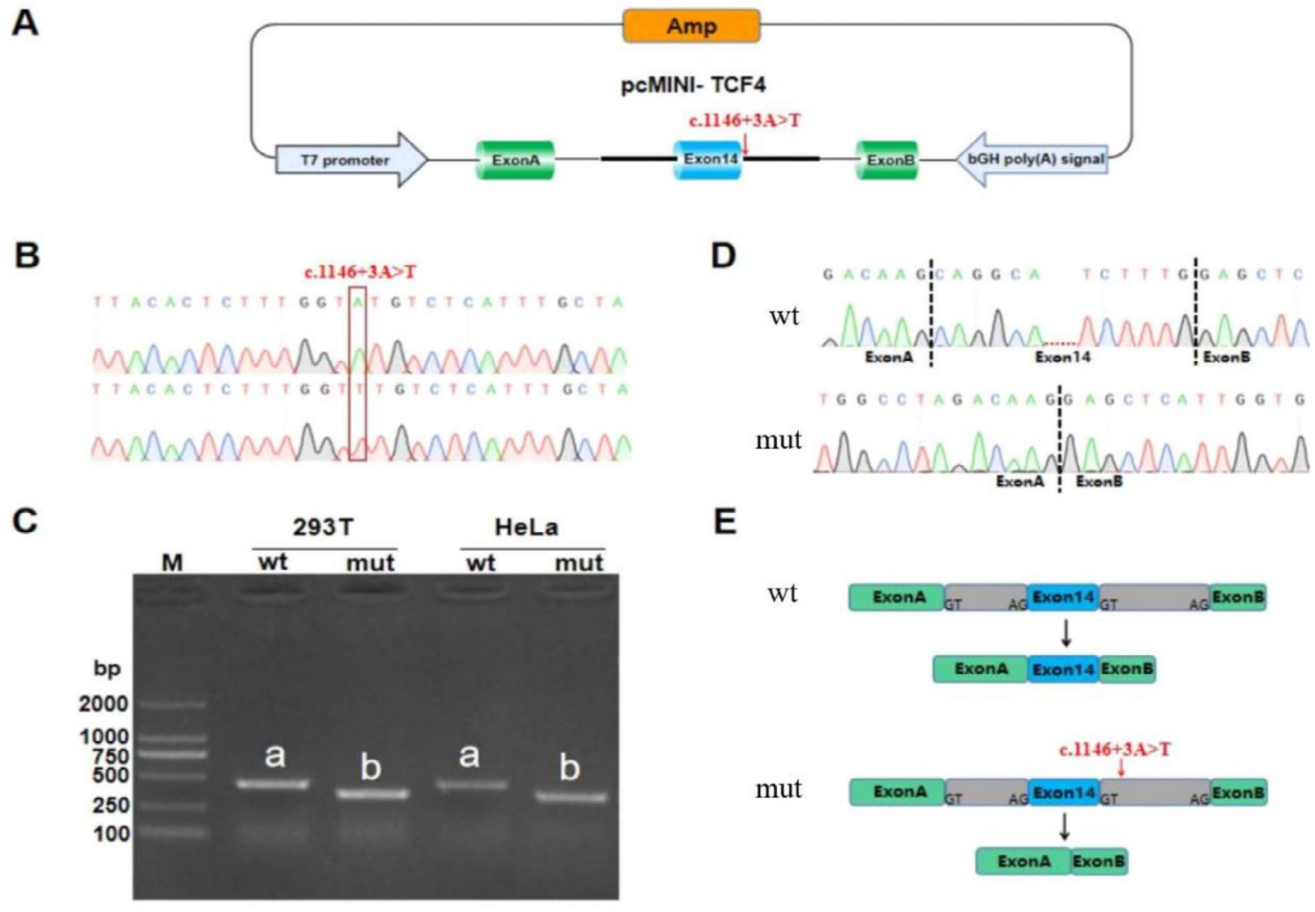
The pcMINI vector detection results. (A) The pcMINI Vector construct scheme; (B) The Sanger sequencing results of pcMINI plasmid used in minigene assays. The c.1146+3 locus was highlighted by a rectangle; (C) The agarose gel electrophoresis image from RT‐PCR transcription analysis shows distinctive bands generated from wild‐type (pcMINI‐wt) or mutant (pcMINI‐mut) constructs transfected in 293T or HeLa cells, respectively; wt, pcMINI‐wt; mut, pcMINI‐mut; (D) The Sanger sequencing result shows exon 14 skipping or wild‐type transcription, respectively, in 293T cells transfected with pcMINI‐wt or pcMINI‐mut; (E) A schematic picture shows distinctive splicing patterns in minigene assay.

After the plasmids of wild‐type and mutant pcMINI‐*TCF4* were successfully constructed (Figure 2A), Sanger sequencing confirmed successful insertion of the target wild‐type or mutant fragment into the pcMINI‐wt and pcMINI‐mut constructs, respectively (Figure 2B). In both HeLa and 293T cells transfected with the wild‐type construct, RT‐PCR analysis revealed a single band of the expected size. In contrast, cells transfected with the mutant construct produced a single, smaller band (Figure 2C). Sequencing of the RT‐PCR products demonstrated that pcMINI‐wt represented normal splicing, consistent with the transcript structure ExonA (192 bp)‐Exon 14 (77 bp)‐ExonB (57 bp). In contrast, pcMINI‐mut corresponded to aberrant splicing characterized by complete skipping of exon 14, resulting in a transcript composed of ExonA (192 bp)‐ExonB (57 bp) (Figure 2D). These findings indicate that the c.1146+3A>T variant disrupts normal *TCF4* pre‐mRNA splicing, leading to exon 14 skipping (Figure 2E). At the transcript level, exon 14 skipping leads to a c.1070_1146 deletion, resulting in a frameshift and subsequently a premature termination codon in exon 15 (p.Gly358Lysfs*4). This alteration is predicted to produce a truncated protein consisting of 360 amino acids.

### 3.4 Variant Pathogenicity Assessment

According to the American College of Medical Genetics and Genomics (ACMG) guidelines for the interpretation of sequence variants, the *TCF4* c.1146+3A>T variant was classified as pathogenic (PS1_Moderate+PS2_Moderate+PM2_Supporting+PP3 + PS3), based on the following evidence: (1) PS1_Moderate: A different nucleotide substitution at the same donor splice site (c.1146+3A>G) has previously been reported as a de novo variant in a patient with Pitt–Hopkins syndrome and was classified as likely pathogenic (Huang et al. 2023); (2) PS2_Moderate: The c.1146+3A>T variant occurred de novo in the fetus, with confirmed parental non‐carrier status; however, the prenatal phenotype was not specific to a single monogenic disorder; (3) PM2_Supporting: The variant is absent from the gnomAD database [v2.1]; (4) PP3: Three independent splicing prediction tools consistently indicated that the variant disrupts the highly conserved donor splice site of exon 14, a non‐phase exon, leading to exon skipping and generation of a prematurely truncated transcript; (5) PS3: Functional evidence from the in vitro minigene assay demonstrated that the variant causes exon 14 skipping, resulting in a frameshift and premature termination codon in exon 15, thereby producing a truncated protein.

## 4 Discussion

A growing body of evidence suggests that increased NT is associated with adverse pregnancy outcomes. In a cohort study of 503 twin pregnancies, the presence of increased NT in at least one fetus was associated with a significantly higher risk of adverse outcomes compared with twin pregnancies in which both fetuses had normal NT measurements (Li et al. 2022). Furthermore, a study of 1658 fetuses with increased NT followed 1309 live births and reported that, even after excluding chromosomal abnormalities and pathogenic or likely pathogenic copy number variants, 1.0% of live‐born infants still exhibited neurodevelopmental delay or abnormalities (Huang et al. 2023). Thus, it is critical to use techniques such as CES to further examine whether there are any genetic alterations, especially SNVs, associated with an elevated NT and, subsequently, any structural anomalies, miscarriage and/or diseases onset after live birth.

Some neurodevelopmental syndromes have been linked with increased NT. For example, Hui et al. identified a de novo heterozygous *SMAD4* variant (c.1499T>C; p.Ile500Thr) in a fetus with NT a measurement of 5.7 mm, resulting in a Myhre syndrome involving multi‐system (Hui et al. 2023). Similarly, Atallah et al. reported two fetuses with increased NT that harbored compound heterozygous variants in the *NUP107* gene and were associated with renal anomalies and neurodevelopmental impairment (Atallah et al. 2025).

PTHS is a severe neurodevelopmental disorder characterized by intellectual disability, impaired sensorimotor gating, language delay, mild‐to‐severe motor developmental delay, and hypotonia in children (Zhao, Wu, et al. 2024). PTHS is primarily caused by *TCF4* gene mutation. However, a direct association between increased NT and *TCF4* gene mutation or PTHS has not been reported.

To date, a wide spectrum of *TCF4 gene* mutations has been identified in several hundred individuals with PTHS, including missense, nonsense, splice variants, small insertions or deletions causing frameshifts, and larger structural rearrangements encompassing part or all of the gene (Aldeeri and Abu‐El‐Haija 2023). In the present study, we specifically focused on splicing variants of *TCF4*, which have been reported relatively infrequently in the literature (Zweier et al. 2008, 2007; de Pontual et al. 2009; Marangi et al. 2011; Zhao, Yang, et al. 2024; Sparber et al. 2020; Jiang et al. 2024). We summarized these cases of *TCF4* splicing variants (Table 1). Notably, these patients consistently exhibited the core clinical features of PTHS, including global developmental delay, severe language impairment, hypotonia, facial dysmorphism, and intellectual disability (Zweier et al. 2008, 2007; de Pontual et al. 2009; Marangi et al. 2011; Zhao, Yang, et al. 2024; Sparber et al. 2020; Jiang et al. 2024). Taken together, these findings support the biological plausibility of the present c.1146+3A>T variant and indicate that its pathogenic effect is consistent with the established mechanism of *TCF4* splicing variation.

**TABLE 1:** Clinical findings in patients with TCF4 splicing variants.

Patient	Sex	Age at report	TCF4 variant	Inheritance	PTHS face	Developmental delay	Intellectual disability‌	Language impairment	Hypotonia	Stereotypies	Palmar creases	Happy disposition	References
P1	F	29 years	c.656‐1G>C	NR	+	+	+	+	+	+	SPC	+	Zweier et al. (2007)
P2	F	18 months	c.655+2insGT	de novo	+	+	+	+	+	+	SPC	+	Zweier et al. (2008)
P3	M	35 months	c.1146+3A>G	de novo	+	+	+	+	+	−	SPC	+	Zweier et al. (2008)
P4	NR	NR	c.923‐2A>G	de novo	+	+	+	+	+	+	−	−	de Pontual et al. (2009)
P5	NR	NR	c.1146+1G>A	de novo	+	+	+	+	+	+	−	NR	de Pontual et al. (2009)
P6	F	7 years 4 months	c.550–1 G>C	de novo	+	+	+	+	+	NR	−	−	Marangi et al. (2011)
P7	NR	NR	c.922+5G>A	NR	+	+	+	+	+	−	−	−	Sparber et al. (2020)
P8	F	16 months	c.1452+3A>G	de novo	+	+	+	+	+	+	−	−	Jiang et al. (2024)
P9	M	4 years 3 months	*c.1A>G	de novo	+	+	+	+	+	+	SPC	+	Zhao, Wu, et al. (2024), Zhao, Yang, et al. (2024)

*Note:* The asterisk in the table indicates the 3'UTR region downstream of the stop codon.

Abbreviations: NR, not report; SPC, single palmar crease.

In our study, the main mechanistic implication of the c.1146+3A>T variant is that it causes complete skipping of exon 14, which leads to a frameshift and a premature truncation. Notably, a similar substitution at the same splice site (c.1146+3A>G) has been reported to produce comparable effects (Zweier et al. 2008). The predicted truncated protein lacks the C‐terminal region of TCF4, including the conserved basic helix–loop–helix (bHLH) domain required for DNA binding and dimerization (Sepp et al. 2012). Loss of this domain is expected to severely impair transcriptional activity. Previous functional studies have demonstrated that PTHS‐associated variants disrupt protein stability, DNA binding, and transactivation capacity, particularly when affecting the bHLH‐containing region (exons 9–19) (Sepp et al. 2012; Bedeschi et al. 2017). Therefore, the molecular consequence of c.1146+3A>T is predicted to be a typical pathogenic loss‐of‐function effect and lead to PTHS.

*TCF4* is broadly expressed and participates in multiple developmental pathways beyond the nervous system. Research reports have indicated that the expansion of CTG trinucleotide repeats (CTG18.1) in the intron of *TCF4* leads to Fuchs endothelial corneal dystrophy (Fautsch et al. 2021). Many apparently non‐neurological features of PTHS, especially abnormal breathing and constipation, may in fact reflect autonomic nervous system dysfunction rather than isolated defects of unrelated organs (Zollino et al. 2019). Variability in clinical presentation has also been reported. For example, Aldeeri et al. reported three familial cases carrying a missense variant in exon 18 (c.1849G>A; p.Val617Ile) who presented with mild, nonspecific neurodevelopmental impairment rather than classical PTHS (Aldeeri and Abu‐El‐Haija 2023). This reveals that patients with *TCF4* variants not only exhibit clinical symptoms of non‐neurological phenotypes but also show significant phenotypic heterogeneity among individuals. This is an aspect that requires special attention in the clinical diagnosis and genetic counseling for PTHS.

We conducted two follow‐ups after the birth of the fetus during the first and second years of its life respectively. We found that fetus A was healthy and normal as expected. In contrast, fetus B showed lateral ventricle widening and delayed growth and development. Widening of the lateral ventricles and growth retardation are typical symptoms of PTHS, providing us evidence for diagnosing fetus B with a potential PTHS. However, since PTHS generally onset during childhood, more symptoms still take time to occur and we will follow up this case closely in the future. In conclusion, both the de novo occurrence of the variant and the direct functional evidence from the minigene assay together provide strong support that c.1146+3A>T is a pathogenic *TCF4* splice‐site variant. And increased NT may be considered a defining prenatal marker of *TCF4*‐related disease. Our research expands the spectrum of prenatal presentations associated with *TCF4* and suggests that PTHS or other *TCF4*‐related disorder should be considered in the differential diagnosis of unexplained increased NT.

## Author Contributions

**Wenlong Shen:** data collection, draft an article. **Yan Zhang:** write a paper, experiment implementation. **Junjie Wu:** revise an essay. **Jue Zhao:** data analysis. **Yaer Lv:** obtain clinical data. **Xiaohua Tang:** design experiments, conduct research guidance, and financial support.

## Funding

This work was supported by the Natural Science Foundation of Zhejiang Province, LY22H160039.

## Ethics Statement

This study was reviewed and approved by the Medical Ethics Committee of Zhejiang Provincial People's Hospital on May 12th, 2025 (Approval Number: QT2025105).

## Consent

Written informed consent was obtained from the parents of the fetus.

## Conflicts of Interest

The authors declare no conflicts of interest.

## Data Availability

The data that support the findings of this study are available on request from the corresponding author. The data are not publicly available due to privacy or ethical restrictions.
